# Bictegravir/Tenofovir Alafenamide/Emtricitabine in Real-Life: A Multi-Year Experience

**DOI:** 10.3390/jcm15145553

**Published:** 2026-07-15

**Authors:** Anna Gidari, Elena Baranello, Carlo Pallotto, Sabrina Morosi, Chiara Papalini, Giulia Gamboni, Lisa Malincarne, Daniele Rosignoli, Maria Carolina Benvenuto, Giacomo Gonnelli, Claudio Ucciferri, Giuseppe Vittorio De Socio

**Affiliations:** Department of Medicine, Clinic of Infectious Diseases, “Santa Maria Della Misericordia” Hospital, University of Perugia, 06132 Perugia, Italylisa.malincarne@unipg.it (L.M.); giuseppe.desocio@unipg.it (G.V.D.S.)

**Keywords:** HIV, bictegravir, Integrase inhibitors, real-life, antiretroviral

## Abstract

**Introduction:** Long-term real-world data on bictegravir/emtricitabine/tenofovir alafenamide (BIC-STR), particularly in key subgroups, remain limited. The primary endpoints of the study were changes in CD4+ cell count and viral load after BIC-STR introduction. The secondary endpoints were the impact of the therapy on metabolic profile and body mass index. **Methods:** Retrospective observational cohort study including people living with HIV (PLWH) receiving BIC-STR with more than 6 months of follow-up at a tertiary Infectious Diseases clinic (Perugia, Italy). Immunovirological outcomes (HIV-RNA, CD4 count, CD4/CD8 ratio) and metabolic parameters were assessed up to 48 months, with subgroup analyses (treatment-naïve/experienced, >60 years, women, and foreign nationals). **Results:** 425 patients were included (median follow-up 37.7 months, IQR, 22.1–55.8 months). Most were treatment-experienced (367/425, 86.4%) and viral suppression improved after switching, reaching 90.7% with HIV-RNA < 50 cp/mL; HIV-RNA declined mainly within the first 6 months and then remained stable. CD4 count and CD4/CD8 ratio increased significantly over time, with similar immunovirological trends in older patients (>60 years; 138/425, 32.3%), women (94/425, 22.1%), and foreign nationals (143/425, 33.7%). In treatment-experienced patients not on statins, HDL and LDL did not change significantly, while triglycerides decreased (median from 110 mg/dL, IQR 78.0–164.0, to 103 mg/dL, IQR 70.3–147.8; *p* = 0.007). In ART-naïve patients (58/425, 13.6%), HDL (median from 41, IQR, 33.0–47.0 to 47, IQR 39.0–54.7, *p* = 0.0003) and BMI (median from 23.2, IQR 21.7–26.3 to 25.7, IQR 23.4–29.0, *p* < 0.0001) increased, whereas other lipid changes were not significant. BMI increased only in underweight and normal-weight naïve patients, not in overweight/obese individuals. **Conclusions:** BIC-STR showed sustained effectiveness, immunological benefit, and favourable metabolic outcomes in routine practice, including, PLWH > 60 years, women, and foreign nationals subgroups.

## 1. Introduction

The evolution from early monotherapy to modern antiretroviral therapy (ART) has transformed HIV from a frequently fatal disease into a manageable chronic condition [1]. Bictegravir is a second-generation integrase strand transfer inhibitor (INSTI) that represents a significant advancement in antiretroviral therapy, offering potent antiviral activity, a high genetic barrier to resistance, once-daily unboosted dosing, minimal drug–drug interactions, and availability as a complete single-tablet regimen with emtricitabine and tenofovir alafenamide (B/F/TAF, marketed as Biktarvy) [2].

Clinical trials have shown the non-inferiority of the bictegravir single-tablet regimen (BIC-STR) to different regimens in both treatment-experienced and naïve patients and these data have been confirmed in real-life studies [3,4,5,6,7,8,9,10].

This study is the result of a cohort observation that lasted 6 years. Our group previously published an observational study on a cohort of people living with HIV (PLWH) on ART with BIC-STR followed by the Clinic of Infectious Diseases of Santa Maria della Misericordia Hospital, Perugia, Italy [7].

The aim of this update is to confirm the durability, clinical efficacy and the metabolic impact of BIC-STR with a longer follow-up period and to focus on specific subgroups that may have different characteristics and responses to a specific therapy.

In particular, the PLWH population is fortunately ageing; therefore, it is fundamental for clinicians to establish the adequacy of the therapy especially in frail population such as the elderly (age > 60 years). Furthermore, although women living with HIV (WLWH) are more than half of PLWH in the world, they are often unrepresented in clinical study both in clinical trials and real-life studies. This disparity is primarily driven by the fact that real-world evidence originates predominantly from European cohorts, where the HIV epidemic is concentrated among males. For instance, according to national surveillance data in Italy, males accounted for 79.2% of all new HIV diagnoses in 2024. Consequently, this regional demographic skew limits the generalizability of real-world data to the global female population [11]. Another important issue is human migration, which may significantly influence the treatment adherence and care continuity in PLWH. For this reason, this study also aimed to analyse the subgroups of elderly, WLWH and foreign-born PLWH in therapy with BIC-STR.

## 2. Materials and Methods

### 2.1. Patients and Data Collection

This observational, retrospective cohort study evaluated PLWH receiving ART with BIC-STR followed by the Clinic of Infectious Diseases of Santa Maria della Misericordia Hospital of Perugia, Perugia, Italy. Eligible PLWH in therapy with BIC-STR from September 2019 to September 2025 were enrolled in the study. The study was approved by the local Ethics Committee (Comitato Etico Regionale—Umbria, protocol number 27300/23/ON—24 May 2023; Registro CER N.: 4551/2023) and conducted according to the Declaration of Helsinki. All patients provided written informed consent for the use of their anonymized data at the time of admission to our department.

The only exclusion criterion was a follow-up duration of less than 6 months after initiation of BIC-STR. In the durability analysis, patients who discontinued BIC-STR before completing 6 months of follow-up were included. Data were extracted from electronic medical records (Netcare^®^, Healthware, Salerno, SA, Italy) and analysed in an anonymized form.

Similarly to our previous study on this topic [7], the following clinical and demographic information was collected for each patient: demographic characteristics (age, sex), HIV risk factors, Centers for Disease Control and Prevention (CDC) classification, history of opportunistic infections, comorbidities, pre-BIC antiretroviral regimen and its duration, metabolic profile (including statin therapy), body mass index, plasma HIV-RNA levels, CD4+ T-cell count, and the CD4/CD8 ratio at different time-points (6, 12, 24, 36 and 48 months).

The primary endpoints of the study were changes in CD4+ cell count and viral load following the BIC-STR introduction. The secondary endpoints included the impact of the therapy on the metabolic profile, the immunological recovery (defined as CD4 cell count > 500 cell/mm^3^), and on the residual viremia.

Effectiveness was defined as HIV-RNA < 50 copies/mL and residual viremia was defined as detectable HIV-RNA (>20 copies/mL) that did not fulfil the criteria for virological failure. Virological failure was defined as two consecutive HIV-RNA measurements > 50 copies/mL obtained at least 1–2 months apart [12,13].

### 2.2. Statistical Analysis

Descriptive variables were summarised as counts and percentages for categorical data, and as mean ± standard deviation (SD) or median with interquartile range (IQR) for continuous data, as appropriate. The cohort was stratified into treatment-naïve versus treatment-experienced patients, older patients (cut-off: 60 years), women, and foreign nationals.

In addition, to confirm the findings of our previous study on this cohort [7], we updated the evaluation of variables associated with favourable immunological recovery (CD4 > 500 cells/mm^3^) and with residual viremia. Based on clinical relevance, the following variables were selected: age, sex, time on BIC-STR, history of opportunistic infections, presence of ≥2 comorbidities, HCV and HBV coinfections, nadir CD4 count, and zenith plasma HIV-RNA.

Univariate analyses were performed using the chi-square test, Fisher’s exact test, paired or unpaired *t*-test, Wilcoxon signed-rank test, or Mann–Whitney U test, as appropriate. Wilcoxon signed-rank and Mann–Whitney U tests were applied when data were not normally distributed or when variances were unequal. Multivariate logistic regression models included variables that reached statistical significance in the univariate analysis (*p* < 0.05). Results are reported as odds ratios with 95% confidence intervals (CI) and *p*-values. Model fit was assessed using the Hosmer–Lemeshow test.

Statistical analyses were performed using GraphPad Prism version 8.3. A *p*-value < 0.05 was considered statistically significant.

## 3. Results

A total of 468 patients received BIC-STR at our centre during the study period; of these, 43 were excluded from the main analysis because their follow-up was less than 6 months. Among these excluded patients, three discontinued BIC-STR therapy due to adverse events and two died. Furthermore, nine patients were lost to follow-up. All patients were included in the durability analysis (*N* = 468). The median follow-up on BIC-STR for the overall cohort was 3.1 years (interquartile range, IQR 1.8–4.6 years).

Overall, 425 PLWH were included (Table 1). Most were male (331/425, 77.9%) and Italian (300/425, 77.9%), with a median age of 53.4 years (IQR 43.9–63.2; range 15.8–87.7). The comorbidity burden was substantial: 320/425 (75.3%) had ≥2 comorbidities. Dyslipidaemia was the most frequent (198/425, 46.6%), followed by hypertension (126/425, 29.6%). Obesity was observed in 93 patients (21.9%), and the median body mass index (BMI) was 25.0 kg/m^2^ (IQR 22.6–27.7). HCV coinfection was reported in 84 patients (19.8%), although only 7 (1.6%) had detectable HCV-RNA at the time of the last observation. HBV coinfection was present in 121 patients (28.5%), and 49 (11.5%) were HBsAg-positive at the most recent assessment.

The prevalence of latent tuberculosis infection (LTBI) was higher among foreign nationals (18/143, 12.5%) than in the overall cohort (26/425, 6.1%). Among the 26 patients diagnosed with LTBI, 20 initiated preventive therapy with isoniazid (the remaining 6 patients either declined or had poor compliance). Seven of these patients received isoniazid concomitantly with BIC-STR for a median duration of 9.5 months (IQR 9–12). Concomitant treatment was well tolerated, with only one patient experiencing a relevant adverse event (elevated transaminases), and no other significant drug–drug interactions were observed.

The median nadir CD4 count was 187.5 cells/mm^3^ (IQR 57.8–344.5 cells/mm^3^), and the median HIV-RNA zenith was 170,000 cp/mL (IQR 52,050–709,750 cp/mL). A nadir CD4 < 200 cells/mm^3^ was observed in 213/425 (50.1%) individuals; among these, 128/213 (60.1%) had a prior AIDS diagnosis. The most common opportunistic infections of the study population before initiating ART were Pneumocystis jirovecii pneumonia (40/128, 31.3%), Kaposi sarcoma (23/128, 18.0%), neurotoxoplasmosis (16/128, 12.7%), and oesophageal candidiasis (15/128, 11.8%). Notably, 23/128 (18.0%) had a history of two or more opportunistic infections.

Genotypic resistance testing prior to BIC-STR initiation was available for 307/425 patients (72.2%). No mutations affecting INSTI activity were detected. The M184V mutation was identified in 9/307 (2.1%), while additional mutations were reported in 88/307 (28.7%).

Most participants were treatment-experienced (367/425, 86.4%), with a median ART duration of 11 years (IQR 5–16). Before switching to BIC-STR, regimens most commonly included INSTIs (267/367, 72.8%), followed by PIs (58/367, 15.8%) and NNRTIs (48/367, 13.1%). Among individuals on prior INSTI-based therapy, the distribution of specific drugs was as follows: elvitegravir (47.9%), dolutegravir (39.7%), raltegravir (12.0%), and cabotegravir (0.4%). Regarding backbone therapy, 240/367 (65.4%) were receiving TAF, 43/367 (11.7%) abacavir (ABC), and 26/367 (7.0%) were on dual therapy. Among PLWH receiving prior dual therapy (*N* = 26), the reasons for switching to BIC-STR were virological concerns for 11 patients (42.3%), regimen simplification for 7 (26.9%), and clinical choice for the remaining 8 individuals (30.8%).

**Figure 1:**
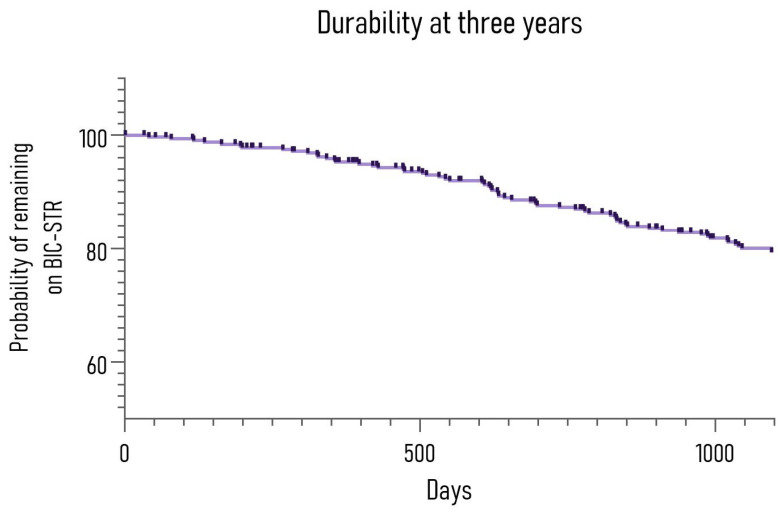
Kaplan–Meier curve showing the probability of remaining on bictegravir/emtricitabine/tenofovir alafenamide single-tablet regimen (BIC-STR) over 3 years of follow-up (*N* = 325). Discontinuation of BIC-STR for any reason was considered the event; patients were censored at last available follow-up.

During follow-up, 55/468 (11.8%) of patients were lost to follow-up or transferred care and 100/468 (21.4%) patients discontinued therapy. Among these, 63/468 (13.5%) switched to dual therapy for regimen simplification and 18/468 (3.8%) died. Discontinuation was mainly attributed to adverse events (12/468, 2.6%) and virological failure (8/468, 1.7%), while 5/468 (1.1%) discontinued due to drug–drug interactions or other contraindications. Neurological adverse events were the most frequent (9/12, 75.0%) reasons for discontinuation, including sleep disturbances, anxiety, and headache; other reported events included myalgia, prurigo nodularis, and renal impairment. Figure 1 depicts the Kaplan–Meier estimates of treatment persistence on BIC-STR over a three-year follow-up period (*N* = 325). To evaluate changes subsequent to BIC-STR initiation, treatment-naïve and treatment-experienced patients were analysed separately. In the treatment-experienced subgroup (*N* = 367), the median follow-up was 3.2 years (IQR 2.0–4.7). A significant improvement in CD4+ count (absolute and percentage) and in the CD4+/CD8+ ratio was observed (Table 2; *p* < 0.0001). Viral suppression also increased substantially: the proportion with HIV-RNA < 50 cp/mL rose from 223/365 (60.7%) before switching to 333/367 (90.7%) after switching. Among patients already suppressed to <50 cp/mL pre-switch, the median duration of suppression was 5.5 years (IQR 2.9–10.3). Using the stricter threshold of <20 cp/mL, 189/367 (51.5%) patients were suppressed at baseline, increasing to 286/367 (76.0%) after the switch (*p* < 0.0001).

**Figure 2:**
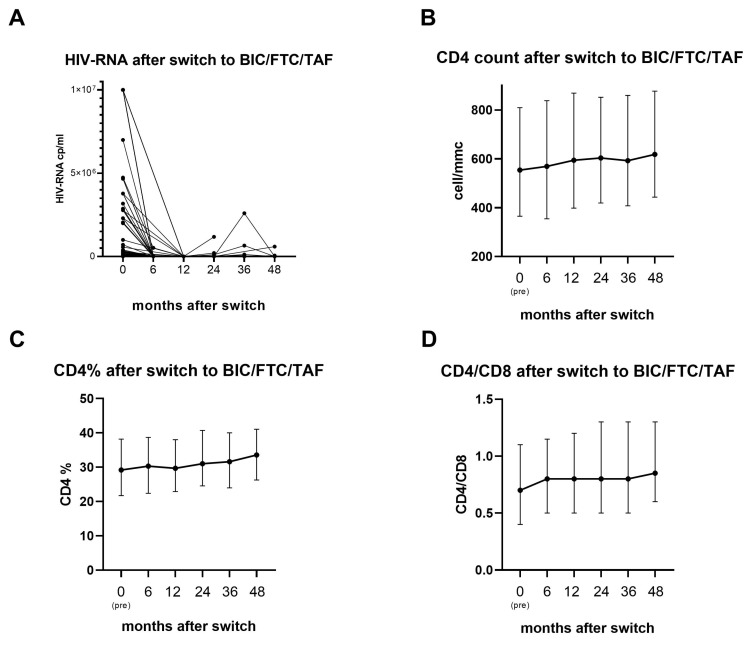
Immunovirological trends in treatment-experienced patients from switch to 48 months: (**A**) HIV-RNA (cp/mL); (**B**) CD4 count (cell/mm^3^); (**C**) CD4%; (**D**) CD4/CD8 ratio.

In particular, when examining trends over time, HIV-RNA levels (Figure 2A) decreased significantly up to 6 months and then remained stable thereafter (*p* < 0.0001). Furthermore, among the 166 patients who had a 48-month follow-up period, 159 (95.7%) had HIV-RNA < 50 cp/mL and 146 (88.0%) had HIV-RNA < 20 cp/mL. CD4 counts showed a progressive increase up to 48 months, both as absolute values (Figure 2B, *p* < 0.0001), as percentages (Figure 2C, *p* < 0.0001), and in terms of the CD4/CD8 ratio (Figure 2D, *p* < 0.0001).

To identify factors potentially associated with incomplete virological suppression—defined as detectable HIV-RNA (>20 cp/mL)—we evaluated several candidate variables, including age, sex, duration of BIC-STR therapy, duration of ART prior to switching, history of opportunistic infections, HCV or HBV coinfections, presence of more than one comorbidity, nadir CD4 count, HIV-RNA zenith, and prior ART regimens before BIC-STR initiation. In univariate analysis, age, time on BIC-STR, nadir CD4 count, and HIV-RNA zenith were significantly associated with incomplete virological suppression [7]. However, at the multivariate logistic regression, none showed significant association to this outcome.

In the treatment-experienced population, metabolic parameters were analysed only in patients not receiving statin therapy, and no statistically significant changes were observed in HDL and LDL levels. Triglycerides, however, showed a statistically significant reduction, decreasing from a median baseline value of 110 mg/dL (IQR 78–164 mg/dL) to 103 mg/dL (IQR 70–147 mg/dL) at the last follow-up (*p* = 0.007) (Table 2).

Stratifying treatment-naïve patients into underweight (BMI < 18.5), normal weight (BMI 18.5–25), and overweight/obese (BMI > 25) groups, BMI showed a significant increase only in patients who were underweight (*p* = 0.05) or normal weight (median of the differences 0.44, CI 0.02–0.67, *p* = 0.0045) at baseline, but not in those who were overweight/obese (*p* = 0.3956) (Table 2). However, given the limited sample size of patients with a BMI < 18.5 (*N* = 9), the findings regarding BMI increase remain descriptive rather than conclusive.

Immunovirological and metabolic changes after BIC-STR initiation were also assessed in ART-naïve patients (*N* = 58), with a median follow-up of 2.4 years (IQR 1.0–3.6 years). As expected, a significant improvement in both virological and immunological parameters was observed (Table 3). Most patients achieved virological suppression, with 37/58 (63.7%) reaching HIV-RNA <20 copies/mL and 48/58 (82.7%) reaching HIV-RNA < 50 copies/mL. Immunological recovery was also favourable, with a median CD4 count of 476 cells/mm^3^ (IQR 325–768 cells/mm^3^), a median CD4 percentage of 30.2% (IQR 21.1–36.2), and a median CD4/CD8 ratio of 0.80 (IQR 0.5–1.1) (Table 3). In this subgroup, increases in HDL cholesterol and BMI were observed, whereas changes in total cholesterol, LDL, triglycerides, and HDL were not statistically significant.

**Figure 3:**
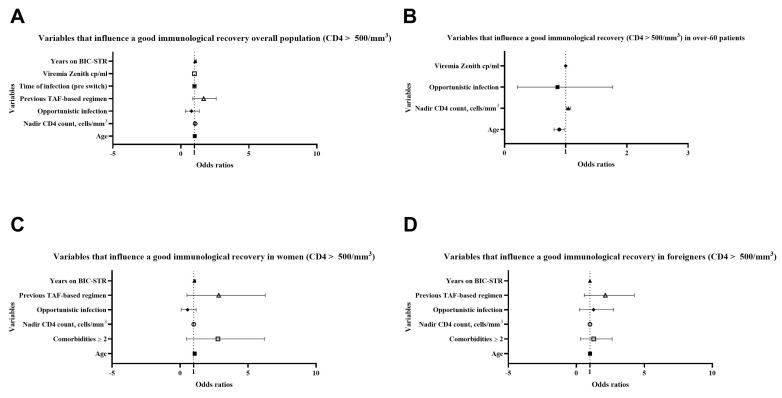
Multiple logistic regression: variables that influence a good immunological recovery, defined as CD4+ T cell count > 500 cell/mm^3^ in overall population (**A**), patients older than 60 years old (**B**), women (**C**) and foreign nationals (**D**).

Factors associated with favourable immunological recovery—defined as a CD4 count >500 cells/mm^3^—were then explored in the overall population. The following variables were included in univariate analyses: age, sex, duration of BIC-STR therapy, duration of ART prior to switching, history of opportunistic infections, HCV or HBV coinfections, presence of more than one comorbidity, nadir CD4 count, and HIV-RNA zenith [7]. Variables entered into the multivariable logistic regression model were as follows: previous ART with TAF, nadir CD4 count, HIV-RNA zenith, previous opportunistic infections, HCV coinfection, and time on BIC-STR. Of these, only the nadir CD4 count was independently associated with immunological recovery (OR 1.005, 95% CI 1.003–1.007) (Figure 3A).

**Over 60 years**

Within our cohort, 138 (32.3%) patients were aged > 60 years. The demographic characteristics of this population are reported in Table 1. At the time of diagnosis, 50 (36.2%) patients had experienced one or more opportunistic infections, distributed as follows: Kaposi sarcoma in 5 (10%) patients, Pneumocystis pneumonia in 12 (24%), cerebral toxoplasmosis in 6 (12%), Candida esophagitis in 5 (1%), disseminated CMV disease in 3 (6%), lymphoma in 3 (6%), disseminated TB in 1 (2%), organ-localised CMV disease in 1 (2%), and progressive multifocal leukoencephalopathy (PML) in 1 (2%).

In this group, 10 (7.2%) were lost to follow-up or changed hospital, 13 (9.4%) were switched to dual therapy for simplification, 9 (6.5%) discontinued therapy and 9 (6.5%) died.

Overall, reasons for therapy discontinuation or modification included: 9 (29.0%) deaths, 13 (41.9%) simplification to dual therapy (including 2 patients switched to long-acting therapy), 3 (9.7%) adverse events, 3 (9.7%) virological failure, and 3 (9.7%) had contraindications. In addition, 10 (7.2%) patients were lost to follow-up.

Regarding immunovirological parameters, we observed a statistically significant increase in the CD4+ count both in absolute numbers and percentage, as well as in the CD4/CD8 ratio, which progressively increased over time up to 48 months (*p* < 0.001).

In patients aged > 60 years, the median pre-switch CD4+ count was 524.5 cells/mm^3^ (IQR 340.5–792.7), increasing to 557.5 cells/mm^3^ (IQR 393.0–820.0) at the last follow-up.

The median CD4+ percentage increased from 30.0% (IQR 20.8–37.8) pre-switch to 32.5% (IQR 24.3–40.1) at the last follow-up, and the median CD4/CD8 ratio increased from 0.70 (IQR 0.4–1.1) to 0.80 (IQR 0.5–1.5).

Pre-switch undetectable HIV-RNA (<20 cp/mL) was observed in 19 (14.6%) patients compared with 115 (83.3%) patients at the last follow-up. The viremia curve showed a decrease in six months, then remained stable.

We also verified which variables influenced favourable immunological recovery (CD4 count > 500 cells/mm^3^) in older PLWH. Advanced age (OR 0.8952, CI 0.8093 to 0.9779) and with less impact lower nadir CD4 count (OR 1.004, CI 1.001 to 1.008) (Figure 3B). The statistical model was appropriate (Hosmer–Lemeshow test: 13.08, *p* = 0.08).

**Women**

We also performed a sub-analysis in the female population, whose demographic characteristics are reported in Table 1. Females accounted for 94 (22%) patients. Treatment experienced patients were more represented (85/94, 90.4%).

At diagnosis, 34 (36.1%) patients were advanced *naïve* (CDC C stage) and had one or more opportunistic infections, including Kaposi sarcoma in 2 (5.8%), Pneumocystis pneumonia in 14 (11.7%), cerebral toxoplasmosis in 5 (14.7%), cryptococcosis in 3 (8.8%), *Candida* esophagitis in 5 (14.7%), disseminated CMV disease in 1 (3.0%), lymphoma in 1 (3.0%), disseminated TB in 3 (8.8%), organ-localised CMV disease in 1 (3%), and HIV encephalopathy in 1 (3%).

Among the female group, 5 (5.3%) died, 9 (9.6%) switched to dual therapy, and 2 (2.1%) discontinued due to reported adverse events; no patient discontinued due to virological failure or contraindications.

Regarding immunovirological parameters, we observed a statistically significant increase in CD4+ count in absolute value, percentage, and CD4/CD8 ratio, which progressively increased over time up to 48 months (*p* < 0.001). Consistent with the other subgroups, HIV-RNA decreased significantly within the first six months and then remained stable through 48 months. Moreover, all treatment-naïve patients achieved undetectable viremia (<50 cp/mL), as did 76 (89.4%) treatment-experienced patients.

A univariate analysis was also performed to evaluate variables associated with favourable immunological recovery (CD4+ > 500 cells/mm^3^) in women; multivariate analysis did not identify any variable significantly associated with immunological recovery (Figure 3C). The statistical model was appropriate (Hosmer–Lemeshow test: 8.2, *p* = 0.40).

**Foreign group**

We also performed a sub-analysis of the foreign population. Foreign nationals were 143 (33.6%) compared with 282 Italians (66.4%) and were geographically distributed as follows: 38% from Africa, 31% from South America, 21% from Eastern Europe, 6% from Asia, and 4% from other regions.

Demographic characteristics are summarised in Table 1. Forty (31%) had CDC stage C and had one or more opportunistic infections, distributed as follows: Kaposi sarcoma in 4 (10.2%), Pneumocystis pneumonia in 4 (10.2%), pulmonary TB in 6 (15.3%), cerebral toxoplasmosis in 7 (18%), HIV encephalopathy in 2 (5.1%), Candida esophagitis in 2 (5.1%), wasting syndrome in 1 (2.5%), lymphoma in 1 (2.5%), disseminated TB in 5 (12.8%), and PML in 1 (2.5%).

In this group, 6 (4.2%) patients died, 15 (10.5%) switched to dual therapy, 5 (3.5%) discontinued due to reported adverse events, and 2 (1.4%) discontinued due to contraindications; no patient discontinued due to virological failure. Overall, 15 (10.5%) patients were lost to follow-up.

Regarding immunovirological parameters, we also observed in this subgroup a statistically significant increase in the CD4+ count both in absolute number and percentage, and in the CD4/CD8 ratio, which progressively increased over time up to 36 months for the absolute count and up to 48 months for percentage and the CD4/CD8 ratio (*p* < 0.001). The viral load decreased during the first six months of follow-up and then remained stable. We also performed a univariate analysis to evaluate variables associated with favourable immunological recovery (CD4+ > 500 cells/mm^3^) in foreign nationals. Six factors were associated with immunological recovery (*p* < 0.05). Multivariate analysis showed that immunological recovery was weakly influenced by the nadir CD4+ count (OR 1.004, CI 1.001 to 1.008), and by the duration of BIC/TAF/FTC therapy (OR 1.002, CI 1.001 to 1.006) (Figure 3D). The statistical model was appropriate (Hosmer–Lemeshow test: 6.7, *p* = 0.57).

## 4. Discussion

Bictegravir is a second-generation INSTI available as a single-tablet co-formulation with tenofovir alafenamide and emtricitabine. A future combination with lenacapavir, a capsid inhibitor, is expected to become available in the near future [14,15].

A large body of evidence supports the safety and effectiveness of BIC-STR in real-world clinical settings [6,8,10,16]. In our previous analysis of the same cohort, we described the characteristics and outcomes of 270 PLWH receiving BIC-STR, with a median follow-up of 2.5 years [7]. The present study was designed to confirm and extend those findings by increasing the follow-up duration (up to 4 years), expanding the cohort (N = 425), and addressing outstanding clinical questions.

Consistent with our earlier results, we observed a low rate of severe adverse events leading to treatment discontinuation, which occurred in 12/468 (2.6%) cases. These results are consistent with those reported in clinical trials for both naive and experienced patients [4,17]. Moreover, 166 patients reached 48 months of follow-up, further supporting the sustained tolerability of BIC-STR over time.

Other real-life studies consistently showed that bictegravir-containing regimens are well tolerated, with low rates of adverse events leading to discontinuation. The most common adverse events reported in observational cohorts and meta-analyses are mild neuropsychiatric symptoms (such as insomnia, abnormal dreams, headache, and agitation), gastrointestinal complaints (nausea, diarrhoea, flatulence), dermatological reactions (rash), and weight gain. Discontinuation due to toxicity is uncommon, typically occurring in 1–2% of patients at 48 weeks, and overall discontinuation rates are low (3–8%) [10,18,19,20]. In our study, we reported sleep disturbances, anxiety, and headache as adverse events. Other disturbances were myalgia, prurigo nodularis, and renal impairment. In the literature, metabolic and hepatic laboratory changes (such as mild increases in blood glucose or ALT) have been observed in ART-experienced patients, but these are not clinically significant and do not lead to discontinuation [18]. Comparative real-world studies show no significant difference in toxicity-related discontinuation rates between bictegravir-based regimens and other integrase inhibitor-based regimens [19]. A 2025 systematic review examining sex differences in second-generation integrase inhibitors found conflicting data for bictegravir specifically. Only two observational studies met inclusion criteria (2069 participants, 12% women), with one reporting three times higher discontinuation hazards in women (adjusted OR: 3.05) and another showing no sex difference (adjusted HR: 0.78) [21]. The authors noted that 287 of 505 screened articles were excluded due to lack of sex-disaggregated data, highlighting an important knowledge gap [21]. In this study, 3/109 (2.7%, the WLWH excluded from the study were considered to achieve this data) women discontinued the therapy for adverse effects.

Consistent with studies showing that BIC/FTC/TAF is non-inferior to other ART regimens, our cohort also experienced a significant rise in the proportion of PLWH with HIV-RNA <50 cp/mL after switching to BIC-STR, reaching 90.7% [3,8,22,23]. Viral load declined markedly within the first six months post-switch and then remained stable over time. In line with this, Armenia et al. reported that after 96 weeks of BIC-STR, detectable viremia occurred in approximately 5% of patients, with no differences between fully active and partially active regimens; loss of virological control was mainly observed in individuals with prior INSTI failure [24]. Other studies similarly indicate that pre-existing NRTI resistance-associated mutations (e.g., M184V, K65N/R) do not predict virological failure in virologically suppressed patients switching to BIC-STR [9,25]. Furthermore, among treatment-experienced patients, 48.5% exhibited documented resistance to at least one antiretroviral drug. While substantial, this prevalence aligns with rates reported in other Italian cohorts. Notably, within the ICONA cohort, 63% of heavily treatment-experienced individuals presented with documented resistance to at least one antiretroviral class [26]. In our clinical practice, BIC-STR is frequently prescribed to patients with suboptimal adherence due to its high genetic barrier and favourable “forgiveness” [27] profile.

Immunologically, we observed a sustained improvement up to 48 months, with increases in CD4 count (absolute and percentage) and in the CD4/CD8 ratio. This pattern was consistent across the overall cohort and all subgroups (treatment-experienced, treatment-naïve, older patients, women, and foreign nationals) and aligns with previously published real-world evidence showing significant CD4 recovery with BIC-STR [6,8]. The latest real-life evidence indicates that CD4 cell recovery after the introduction of BIC-STR is generally modest but consistent, with significant increases observed particularly in treatment-naïve individuals and certain subgroups. In treatment-naïve people living with HIV, median CD4 increases at 48 weeks have been reported, which is comparable or slightly superior to other integrase inhibitor-based regimens [28].

For patients with persistent low-level viremia, switching to BIC/FTC/TAF has been associated with a significant mean CD4 increase (from 486 to 619 cells/mm^3^) and improvement in CD4/CD8 ratio over 12–24 months [29]. In randomised controlled trials, mean CD4 increases of 180–233 cells/mm^3^ at 48 weeks have been observed, confirming robust immunological recovery [30,31]. Real-world systematic reviews and meta-analyses further support high rates of virologic suppression, with favourable immunological outcomes [20].

In this study, we explored predictors of favourable immunological recovery, defined as a CD4 count >500 cells/mm^3^. In the overall cohort, nadir CD4 count was the only variable associated with this outcome, whereas the association previously observed with a prior TAF-based regimen was not confirmed. In patients aged >60 years, immunological improvement was mainly influenced by age and nadir CD4 count, highlighting the clinical value of early diagnosis and timely ART initiation, particularly in more vulnerable individuals. Notably, despite these constraints, the older subgroup still showed a clear improvement over time in CD4 count, CD4 percentage, and CD4/CD8 ratio.

The most recent comprehensive study on BIC-STR in the oldest population is a 2025 integrated analysis published in BMC Infectious Diseases that evaluated efficacy and safety in people with HIV aged ≥50 years across six Phase 3 clinical trials [32]. The study included 96 treatment-naïve and 450 virologically suppressed participants ≥50 years of age, with follow-up through Week 240 for treatment-naïve patients and Week 48 for virologically suppressed patients [32]. Efficacy outcomes were excellent: virologic suppression was achieved in 98.5% of treatment-naïve participants ≥50 years by week 240, comparable to 98.6% in those <50 years [32]. Among virologically suppressed patients, 93.6% maintained suppression at week 48 in both age groups, with virological failure rates of only 0.9% versus 1.4% in older versus younger participants [32]. CD4 T-cell recovery, adherence rates (≥95%), body weight changes, lipid profiles, renal function, bone health, and treatment-emergent adverse events showed no clinically significant differences between older and younger participants. The incidence of emergent diabetes and hypertension was also similar [32].

Additionally, a 2025 real-world study from Italy specifically examined BIC-STR in patients aged ≥ 50 years [33]. This retrospective cohort of 214 patients (mean age 60.6 years) demonstrated high virological suppression rates at 24 months: 85.7% in treatment-naïve and 93.9% in treatment-experienced patients [33]. A notable finding was significant improvement in the CD4+/CD8+ ratio in experienced patients, normalising from 0.95 to 1.12 [33]. Similar findings were observed in a Spanish real-world study (RETROBIC) where patients ≥60 years showed significant immunological improvements with CD4 cell count increasing and CD8 cell count decreasing but without any change in CD4/CD8 ratio [34].

The pivotal 96-week Phase 3b trial specifically enrolled virologically suppressed adults ≥65 years (N = 86, mean age 70 years, range 65–80) [35]. Results showed 100% maintained viral suppression (no participants had HIV-1 RNA ≥ 50 copies/mL) at weeks 72 and 96, with no treatment-emergent resistance [35].

In older individuals, a low CD4/CD8 ratio has been associated with poorer prognosis. Although the underlying mechanisms remain unclear, it has been hypothesised that reduced CD4 counts may contribute to a state of subtle immunodeficiency, while an expanded population of potentially dysfunctional CD8+ cells may promote a pro-inflammatory environment [36]. Therefore, the CD4/CD8 ratio has been proposed as a useful biomarker in the clinical monitoring of HIV infection.

In our cohort, the prevalence of HBV co-infection among PLWH aged > 60 years was 15.2%. This notably higher prevalence may be largely attributed to a cohort effect; while younger generations have widely benefited from the introduction of universal HBV vaccination campaigns across Europe over the last few decades, older individuals missed these programmes, resulting in a higher historical and lifetime risk of HBV exposure. Globally, HBV co-infection affects 7.6% of people living with HIV, who face 1.42 times higher odds of infection than HIV-negative individuals, with a heavy burden in sub-Saharan Africa [37].

Furthermore, we performed an analysis of the female subgroup because despite women representing more than half of PLWH, it is an understudied population. This population is also underrepresented in our cohort because it is only 22.1% and this is in line with most of the real-life cohorts described in the literature [33,34,38,39]. In this group, after BIC-STR introduction, we observed an improvement of all immune-virological parameters. Evaluating variables that could impact the immunological recovery, none were significantly associated with the outcome. An integrated analysis of 373 women across 5 trials showed that BIC-STR achieved virologic suppression (HIV-1 RNA < 50 copies/mL) at week 48 in ≥95% of virologically suppressed participants and ≥87% of ART-naive participants, with rates similar to comparator regimens [40]. A dedicated phase 3 trial in 470 virologically suppressed women demonstrated noninferiority to staying on baseline regimen, with only 1.7% experiencing virologic failure in both groups [41]. Importantly, no treatment-emergent resistance was detected in any woman receiving BIC-STR across these studies [40,41]. Furthermore, recently, BIC-STR was approved in pregnancy: recent pharmacokinetic studies demonstrate that while bictegravir exposure decreases during pregnancy (total pharmacokinetic AUC reduced by 46–59% and trough concentrations by 62–74% compared to postpartum), drug levels remain above the therapeutic threshold needed for virologic suppression [42,43]. These findings highlight the need for additional real-world data on women receiving BIC-STR.

Foreign patients represent another clinically challenging subgroup, due to factors such as language barriers, incomplete medical history, frequent changes in healthcare providers, and cultural differences. This population is increasing in our centres and warrants focused evaluation. In our cohort, foreign nationals accounted for more than one-third of participants (33.7%). Their immunovirological trajectory was comparable to that observed in the other subgroups. When investigating predictors of immunological recovery, nadir CD4+ count and duration of BIC/TAF/FTC therapy were significantly associated with the outcome.

Metabolic health remains a further key aspect of care in PLWH. Concerns regarding metabolic changes are largely driven by evidence reporting dyslipidaemia following a switch from TDF to TAF [44,45,46,47].

In this study, metabolic parameters were corrected for statin therapy in the treatment-experienced population, and no statistically significant changes were observed in HDL and LDL levels. Triglycerides, however, showed a modest but statistically significant reduction. However, in naïve populations, only HDL cholesterol showed a significant increase.

In ART-experienced patients switching to BIC-STR, real-world data consistently show lipid improvements. An Italian real-life cohort of 539 patients found that at 6 months, ART-experienced individuals switching to BIC-STR showed significant reductions in total cholesterol and triglycerides [18]. A UK study of 135 patients switching to BIC-STR demonstrated that those with the highest baseline total cholesterol and triglyceride quartiles had significant improvements in lipid profiles, particularly those switching from protease inhibitor therapy [48]. Comparative lipid data from a 2025 South Korean study of 194 treatment-naive patients showed that over 2 years, those receiving DTG/3TC had significantly smaller LDL cholesterol increases compared to BIC-STR (regression coefficient β = −8.03, *p* = 0.035), with a trend toward improved Total Cholesterol/HDL ratio (β = −0.19, *p* = 0.094) [49].

In addition, one study reported that after switching to BIC-STR, patients with the poorest baseline lipid profiles experienced a significant improvement, and those discontinuing protease inhibitors showed a reduction in triglyceride levels [48]. Comparable findings were observed in the BICTEL cohort, which documented a significant decrease in the total cholesterol/HDL ratio and LDL levels, alongside a significant increase in BMI across regimens [6].

Conversely, an Italian multicentre cohort published in 2026 reported that, after 48 weeks, SCORE2 cardiovascular risk increased by +0.30 in patients receiving BIC-STR (*p* < 0.001), whereas it decreased in those treated with doravirine/3TC/TDF [50].

In our cohort, BMI increased only among patients who were underweight (BMI < 18.5) or normal weight (BMI 18.5–25) at baseline, with no significant change in overweight/obese individuals. This pattern may reflect a broader recovery of health status rather than a treatment-related adverse effect.

The most recent prospective cohort data demonstrate modest and comparable weight gain between BIC-STR and other INSTI regimens. A 2025 Spanish multicentre study of 680 ART-naive participants found mean weight gain of 1.4 kg (95% CI: 1.1–1.8) at 96 weeks with BIC-STR, with no significant difference compared to DTG + 3TC [51]. Furthermore, there were no differences in BMI changes or incidence of clinical metabolic events (hypertension, diabetes, dyslipidemia, liver steatosis) [51].

A 2026 randomised trial (INSTINCT) comparing switching to BIC-STR versus continuing DTG/3TC in virologically suppressed individuals found weight changes were modest and similar between groups over 96 weeks, with no difference in the proportion experiencing ≥5% weight gain [52]. However, US guidelines note that women and Black individuals are disproportionately affected by INSTI- and TAF-associated weight gain, with female sex associated with 1.5 times the odds of ≥10% weight gain compared to males (17.4% vs. 12.2%) [53].

Importantly, switching strategies to reverse weight gain have been largely unsuccessful. Studies show that discontinuing TAF or switching from INSTI regimens does not significantly reverse weight gain, unless switching to efavirenz (which likely suppresses weight through toxic effects and is not recommended) [53].

Therefore, by analysing lipid parameters adjusted for statin use and stratifying BMI into underweight, normal-weight, and overweight/obese categories, we confirmed and extended our previous findings. Overall, these results suggest that switching to BIC-STR should not be regarded as metabolically unfavourable; rather, it may be associated with potentially beneficial long-term effects, which warrant further investigation in future studies.

A major strength of our study is that the findings derive from real-world clinical practice, reflecting routine use of the regimen and, therefore, potentially offering greater external validity than controlled clinical trials specifically designed to assess drug efficacy. In addition, compared with our previous report, we substantially increased both the sample size and the duration of follow-up. Moreover, the larger sample size allowed a more robust characterisation of three clinically relevant subgroups: older patients, women, and foreign nationals.

The main limitations of this study include its retrospective, single-centre design; the use of BMI, which is not the optimal measure for assessing obesity and fat distribution; and the absence of more specific biomarkers of inflammation and ageing.

## 5. Conclusions

In our cohort, BIC-STR maintained strong efficacy and tolerability over a longer follow-up and in a larger sample of PLWH. These favourable outcomes were consistently observed across all analysed subgroups, including ART-naïve and treatment-experienced patients, older individuals, women, and foreign nationals.

Moreover, the suspected negative metabolic impact was not confirmed in our population, and weight gain appeared to affect patient subgroups differently rather than uniformly.

Overall, these findings support BIC-STR as a reliable option for treatment switch and regimen simplification, as well as for initiating ART, across a broad range of PLWH populations.

## Figures and Tables

**Table 1:** Demographic characteristics of the overall population and the subgroups. Follow-up > 6 months.

Group	Overall Population	Old (>60 Years)	Women	Foreign
*N*	425	138	94	143
Age, median (IQR)	53.4 (43.9–62.1)	64 (62.2–59.4)	49.6 (43.7–56.7)	45.7 (37.1–52.3)
Male, *N* (%)	331 (77.9)	122 (88.4)		81 (56.6)
Italians, *N* (%)	300 (70.5)	121 (87.6)	41 (43.6)	
CDC C, *N* (%)	126 (31.3)	50 (36.2)	34 (36.1)	39 (27.2)
Comorbidities >2, *N* (%)	320 (75.2)	128 (92.7)	66 (70.2)	93 (65.0)
Dyslipidaemia, *N* (%)	198 (46.5)	79 (57.2)	42 (44.6)	54 (37.7)
Statin therapy before	48 (11.3)	34 (24.6)	4 (4.3)	6 (4.2)
Statin therapy after	115 (27.1)	70 (50.7)	17 (18.1)	22 (15.3)
Hypertension, *N* (%)	126 (30.0)	71 (50.0)	23 (20.0)	25 (20.0)
Chronic liver disease, *N* (%)	113 (26.5)	46 (33.3)	20 (21.2)	32 (22.3)
Metabolic syndrome, *N* (%)	39 (9.1)	19 (13.7)	9 (9.5)	7 (4.8)
Obesity, *N* (%)	93 (21.8)	23 (16.6)	37 (39.3)	41 (28.6)
Anxiety depressive syndrome, *N* (%)	49 (11.5)	18 (13%)	11 (11.7%)	13 (9.0)
Neoplasia, *N* (%)	48 (11.2)	26 (18.8)	8 (8.5)	11 (7.6)
DM2, *N* (%)	47 (10.0)	31 (20.0)	9 (10)	22 (15.5)
CAD, *N* (%)	38 (8.9)	22 (16)	3 (3.1)	5 (3.4)
COPD, *N* (%)	29 (7.0)	19 (14)	4 (4.0)	5 (3.0)
CKD, *N* (%)	28 (6.5)	21 (15.2)	4 (4.2)	8 (5.6)
Latent tuberculosis, *N* (%)	26 (6.1)	9 (6.5)	7 (7.4)	18 (12.5)
Stroke history, *N* (%)	14 (3.3)	9 (6.5)	2 (2.1)	0 (0.0)
BMI, median (IQR)	25 (22.5–27.7)	25.2 (23.1–27.7)	24.6 (21.0–28.4)	25.1 (22.4–29.7)
HCV ab positive, *N* (%)	84 (19.7)	38 (27.5)	19 (20.2)	19 (13.3)
HCV RNA detectable, *N* (%)	7 (1.6)	2 (1.4)	2 (2.1)	2 (1.3)
HBsAg positive, *N* (%)	49 (11.5)	21 (15.2)	7 (7.4)	19 (13.2)
Nadir CD4 count cell/mm^3^, median (IQR)	187.5 (57.7–344.7)	129.0 (48.0–292.0)	190 (64.0–306.0)	217 (97.0–353.0)
HIV-RNA Zenith cp/mL, median (IQR)	170,000 (52,050–709,750)	226,000 (49,090–753,000)	222,000 (67,050–568,500)	222,000 (67,050–568,500)
Previous ART (last before Biktarvy), *N* 361:				
INSTI, *N* (%)	267 (62.8)	110 (79.7)	56 (59.6)	80 (56.0)
NNRTI, *N* (%)	48 (11.2)	10 (7.2)	16 (17)	25 (17.4)
PI, *N* (%)	58 (13.6)	17 (12.3)	17 (18)	22 (15.3)
TAF backbone, *N* (%)	240 (56.4)	82 (59.4)	50 (53.1)	78 (54.5)
ABC backbone, *N* (%)	43 (10.1)	26 (18.8)	8 (85.0)	10 (7.0)
Dual-th, *N* (%)	26 (6.0)	11 (8.0)	7 (7.0)	9 (6.0)
Months on ART pre-switch, median, (IQR)	113 (90.4)	148 (90.4)	131 (97.1)	105 (87.2)
Availability of drug-resistance test, *N* (%)	307 (72.2)	105 (76.0)	60 (63.8)	101 (70.6)
Drug resistance (≥ 1), *N* (%)	206 (48.5)	31 (29.5)	29 (29.3)	29 (29.3)
Medical examination *N*/year, median (IQR)	2.3 (1.9–3.1)	2.2 (1.9–6.5)	2.4 (1.9–3.2)	2.5 (2.0–3.2)

CDC, Centers for Disease Control and Prevention; ART, anti-retroviral therapy; INSTI, Integrase inhibitor; NNRTI, Non-Nucleoside Reverse Transcriptase Inhibitor; PI, Protease Inhibitor; TAF, Tenofovir alafenamide; ABC, abacavir; Dual-th, dual-therapy; Chronic Coronary Artery Disease, CAD; DMII, Diabetes mellitus type II; CKD, Chronic Kidney Disease; COPD, Chronic Obstructive Pulmonary Disease; BMI, body mass index; SD, standard deviation; IQR, interquartile range.

**Table 2:** Immuno-virological and metabolic variation after switch to Biktarvy in ART experienced patients (*N* = 367). Cholesterol and triglycerides data were analysed only in patients not in statin therapy.

	Before Switch	Last Value	*p*-Value
CD4+ T cell count, median (IQR)	556 (364.7–806.5)	610 (433.0–867.0)	<0.0001
CD4+%, median (IQR)	29.2 (21.5–37.9)	32.5 (25.0–40.4)	<0.0001
CD4+/CD8+ T cell ratio, median (IQR)	0.70 (0.4–1.1)	0.90 (0.6–1.4)	<0.0001
HIV RNA < 20 cp/mL, *N* (%)	189 (51.5%)	286 (76%)	0.004
HIV RNA < 50 cp/mL, *N* (%)	223 (60.7%)	333 (90.7%)	0.0001
Total cholesterol, median (IQR) [*N* = 255]	197.0 (174.0–229.0)	196.5 (173.0–217.0)	0.079
HDL, median (IQR) [*N* = 255]	49.0 (42.6–58.0)	49.0 (43.0–58.0)	0.4808
LDL, median (IQR) [*N* = 255]	123.0 (97.4–146.2)	120.0 (100.0–142.8)	0.425
Triglycerides, median (IQR) [*N* = 255]	110.0 (78.0–164.0)	103.0 (70.3–147.8)	0.007
BMI, median (IQR)	25.3 (22.8–27.9)	25.4 (22.7–28.7)	0.0065
BMI from <18.5 kg/m^2^, median (IQR) [*N* = 9]	17.7 (16.7–18.4)	18.5 (17.7–20.3)	0.05
BMI from 18.5 to 25 kg/m^2^, median (IQR) [*N* = 145]	23.0 (21.0–24.0)	22.9 (21.5–24.8)	0.0045
BMI from >25 kg/m^2^, median (IQR) [*N* = 184]	28.0 (26.0–30.0)	28. (26.1–31.3)	0.3956

IQR, interquartile range; BMI body mass index; cp, copies; nd, not detectable; *p*-values calculated using the Wilcoxon signed-rank test for paired data.

**Table 3:** Immuno-virological and metabolic variation after Biktarvy introduction in ART naive patients (*N* = 58). Median follow-up 2.4 years (IQR 1.0–3.6).

	Basal Values	Last Values	*p*-Value
CD4+ T cell/mm^3^, median (IQR)	192.5 (53.2–334)	476 (325.0–768.0)	<0.0001
CD4+%, median (IQR)	14.2 (7.3–23.0)	30.2 (21.1–36.2)	<0.0001
CD4+/CD8+ T cell ratio, median (IQR)	0.20 (0.1–0.45)	0.80 (0.5–1.1)	<0.0001
HIV-RNA <50 cp/mL, ***N*** (%) <20 cp/mL, ***N*** (%) Undetectable, ***N*** (%)		48 (82.8) 37 (63.8) 23 (39.7)	
HIV-RNA cp/mL, median (IQR)	732,500 (16,800–2,080,000)	<20 (nr–38)	
Total Cholesterol, median (IQR)	175 (150.2–199.2)	177 (157.0–202.7)	0.21
HDL, median (IQR)	41 (33.0–47.0)	47 (39.0–54.7)	0.0003
LDL, median (IQR)	106.2 (88.1–129.1)	107.4 (84.1–129.0)	0.67
Triglycerides, median (IQR)	101 (81.5–138.2)	96.0 (68.7–137.7)	0.81
BMI, median (IQR)	23.2 (21.7–26.3)	25.7 (23.4–29.0)	<0.0001

IQR, interquartile range; BMI body mass index; cp, copies; *p*-values calculated using the Wilcoxon signed-rank test for paired data.

## Data Availability

The datasets analysed during the current study are available from the corresponding author on reasonable request.

## References

[1] Ahonkhai A.A., Fields E.L., Secord E.A., Golden W.C., Collins-Ogle M.D. (2020). HIV Symposium. J. Natl. Med. Assoc..

[2] Tsiang M., Jones G.S., Goldsmith J., Mulato A., Hansen D., Kan E., Tsai L., Bam R.A., Stepan G., Stray K.M. (2016). Antiviral Activity of Bictegravir (GS-9883), a Novel Potent HIV-1 Integrase Strand Transfer Inhibitor with an Improved Resistance Profile. Antimicrob. Agents Chemother..

[3] Orkin C., DeJesus E., Sax P.E., Arribas J.R., Gupta S.K., Martorell C., Stephens J.L., Stellbrink H.J., Wohl D., Maggiolo F. (2020). Fixed-dose combination bictegravir, emtricitabine, and tenofovir alafenamide versus dolutegravir-containing regimens for initial treatment of HIV-1 infection: Week 144 results from two randomised, double-blind, multicentre, phase 3, non-inferiority trials. Lancet HIV.

[4] Stellbrink H.J., Arribas J.R., Stephens J.L., Albrecht H., Sax P.E., Maggiolo F., Creticos C., Martorell C.T., Wei X., Acosta R. (2019). Co-formulated bictegravir, emtricitabine, and tenofovir alafenamide versus dolutegravir with emtricitabine and tenofovir alafenamide for initial treatment of HIV-1 infection: Week 96 results from a randomised, double-blind, multicentre, phase 3, non-inferiority trial. Lancet HIV.

[5] Molina J.M., Ward D., Brar I., Mills A., Stellbrink H.J., López-Cortés L., Ruane P., Podzamczer D., Brinson C., Custodio J. (2018). Switching to fixed-dose bictegravir, emtricitabine, and tenofovir alafenamide from dolutegravir plus abacavir and lamivudine in virologically suppressed adults with HIV-1: 48 week results of a randomised, double-blind, multicentre, active-controlled, phase 3, non-inferiority trial. Lancet HIV.

[6] Lazzaro A., Cacciola E.G., Borrazzo C., Innocenti G.P., Cavallari E.N., Mezzaroma I., Falciano M., Fimiani C., Mastroianni C.M., Ceccarelli G. (2022). Switching to a Bictegravir Single Tablet Regimen in Elderly People Living with HIV-1: Data Analysis from the BICTEL Cohort. Diagnostics.

[7] Gidari A., Benedetti S., Tordi S., Zoffoli A., Altobelli D., Schiaroli E., De Socio G.V., Francisci D. (2023). Bictegravir/Tenofovir Alafenamide/Emtricitabine: A Real-Life Experience in People Living with HIV (PLWH). Infect. Dis. Rep..

[8] Mazzitelli M., Trunfio M., Putaggio C., Sasset L., Leoni D., Lo Menzo S., Mengato D., Cattelan A.M. (2022). Viro-Immunological, Clinical Outcomes and Costs of Switching to BIC/TAF/FTC in a Cohort of People Living with HIV: A 48-Week Prospective Analysis. Biomedicines.

[9] Tsai M.S., Sun H.Y., Chen C.P., Lee C.H., Lee C.Y., Liu C.E., Tang H.J., Hung T.C., Li C.W., Lee Y.T. (2023). Switching to coformulated bictegravir, emtricitabine, and tenofovir alafenamide maintained viral suppression in adults with historical virological failures and K65N/R mutation. Int. J. Infect. Dis..

[10] Ambrosioni J., Rojas Liévano J., Berrocal L., Inciarte A., de la Mora L., González-Cordón A., Martínez-Rebollar M., Laguno M., Torres B., Ugarte A. (2022). Real-life experience with bictegravir/emtricitabine/tenofovir alafenamide in a large reference clinical centre. J. Antimicrob. Chemother..

[11] Istituto Superiore di Sanità Aggiornamento Delle Nuove Diagnosi Di Infezione Da Hiv E Dei Casi Di Aids In Italia Al 31 Dicembre 2024. https://www.salute.gov.it/new/sites/default/files/2025-11/COA.

[12] Calmy A., Ford N., Hirschel B., Reynolds S.J., Lynen L., Goemaere E., Garcia De La Vega F., Perrin L., Rodriguez W. (2007). HIV viral load monitoring in resource-limited regions: Optional or necessary?. Clin. Infect. Dis..

[13] Ryom L., De Miguel R., Cotter A.G., Podlekareva D., Beguelin C., Waalewijn H., Arribas J.R., Mallon P.W.G., Marzolini C., Kirk O. (2022). Major revision version 11.0 of the European AIDS Clinical Society Guidelines 2021. HIV Med..

[14] Doan J., Brunzo-Hager S., Satterly B., Cory T.J. (2023). Expanding therapeutic options: Lenacapavir + bictegravir as a potential treatment for HIV. Expert Opin. Pharmacother..

[15] Orkin C., Ruane P.J., Hedgcock M., Gaultier C., Losso M.H., Trottier B., Lutz T., O’Reilly M., Bloch M., Slim J. (2026). Switch to single-tablet bictegravir-lenacapavir from a complex HIV regimen (ARTISTRY-1): A randomised, open-label, phase 3 clinical trial. Lancet.

[16] Chen L.-Y., Sun H.-Y., Chuang Y.-C., Huang Y.-S., Liu W.-D., Lin K.-Y., Chang H.-Y., Luo Y.-Z., Wu P.-Y., Su Y.-C. (2023). Patient-reported outcomes among virally suppressed people living with HIV after switching to Co-formulated bictegravir, emtricitabine and tenofovir alafenamide. J. Microbiol. Immunol. Infect..

[17] Sax P.E., Rockstroh J.K., Luetkemeyer A.F., Yazdanpanah Y., Ward D., Trottier B., Rieger A., Liu H., Acosta R., Collins S.E. (2021). Switching to Bictegravir, Emtricitabine, and Tenofovir Alafenamide in Virologically Suppressed Adults With Human Immunodeficiency Virus. Clin. Infect. Dis..

[18] Squillace N., Ricci E., Maggi P., Taramasso L., Menzaghi B., De Socio G.V., Piconi S., Maurizio Celesia B., Orofino G., Sarchi E. (2023). Real-life safety of Emtricitabine/Tenofovir Alafenamide/Bictegravir. PLoS ONE.

[19] Rocabert A., Borjabad B., Berrocal L., Blanch J., Inciarte A., Chivite I., Gonzalez-Cordon A., Torres B., Ambrosioni J., Martinez-Rebollar M. (2023). Tolerability of bictegravir/tenofovir alafenamide/emtricitabine versus dolutegravir/lamivudine as maintenance therapy in a real-life setting. J. Antimicrob. Chemother..

[20] Chivite I., Berrocal L., de Lazzari E., Navadeh S., Lluis-Ganella C., Inciarte A., de la Mora L., González-Cordón A., Martínez-Rebollar M., Laguno M. (2024). Effectiveness, safety and discontinuation rates of bictegravir/emtricitabine/tenofovir alafenamide (BIC/FTC/TAF) in people with HIV using real-world data: A systematic review and meta-analysis. J. Antimicrob. Chemother..

[21] Fang Z., Povshedna T., Patterson R., Ready E., Cote H.C.F., Murray M.C.M., King E.M. (2025). Sex differences in discontinuations due to side effects of second-generation integrase strand transfer inhibitors: A systematic review and meta-analysis. EClinicalMedicine.

[22] Hagins D., Kumar P., Saag M., Wurapa A.K., Brar I., Berger D., Osiyemi O., Hileman C.O., Ramgopal M.N., McDonald C. (2021). Switching to Bictegravir/Emtricitabine/Tenofovir Alafenamide in Black Americans with HIV-1: A Randomized Phase 3b, Multicenter, Open-Label Study. J. Acquir. Immune Defic. Syndr..

[23] Gutiérrez-Lorenzo M., Rubio-Calvo D., Urda-Romacho J. (2021). Effectiveness, safety, and economic impact of the bictegravir/emtricitabine/tenofovir alafenamide regimen in real clinical practice cohort of hiv-1 infected adult patients. Rev. Esp. Quimioter..

[24] Armenia D., Forbici F., Bertoli A., Berno G., Malagnino V., Gagliadini R., Borghi V., Gennari W., Cicalini S., Buonomini A. (2022). Bictegravir/emtricitabine/tenofovir alafenamide ensures high rates of virological suppression maintenance despite previous resistance in PLWH who optimize treatment in clinical practice. J. Glob. Antimicrob. Resist..

[25] Chen G.J., Sun H.Y., Chen L.Y., Hsieh S.M., Sheng W.H., Da Liu W., Chuang Y.C., Huang Y.S., Lin K.Y., Wu P.Y. (2022). Low-level viraemia and virologic failure among people living with HIV who received maintenance therapy with co-formulated bictegravir, emtricitabine and tenofovir alafenamide versus dolutegravir-based regimens. Int. J. Antimicrob. Agents.

[26] Lo Caputo S., Poliseno M., Tavelli A., Gagliardini R., Rusconi S., Lapadula G., Antinori A., Francisci D., Sarmati L., Gori A. (2024). Heavily treatment-experienced persons living with HIV currently in care in Italy: Characteristics, risk factors, and therapeutic options—the ICONA Foundation cohort study. Int. J. Infect. Dis..

[27] Sax P.E., Andreatta K., Molina J.-M., Daar E.S., Hagins D., Acosta R., D’Antoni M.L., Chang S., Martin R., Liu H. (2022). High efficacy of switching to bictegravir/emtricitabine/tenofovir alafenamide in people with suppressed HIV and preexisting M184V/I. AIDS.

[28] Ciccullo A., Baldin G., Borghi V., Oreni L., Lagi F., Fusco P., Giacomelli A., Torti C., Sterrantino G., Mussini C. (2024). Comparing the efficacy and safety of a first-line regimen with emtricitabine/tenofovir alafenamide fumarate plus either bictegravir or dolutegravir: Results from clinical practice. Int. J. Antimicrob. Agents.

[29] Petrakis V., Rafailidis P., Babaka N., Trypsianis G., Maria P., Zisaki S., Papazoglou D., Panagopoulos P. (2026). Bictegravir’s effect on persistent low-level viremia and immunological response. BELAIR study: A pilot prospective observational study. Int. J. STD AIDS.

[30] Gallant J., Lazzarin A., Mills A., Orkin C., Podzamczer D., Tebas P., Girard P.-M., Brar I., Daar E.S., Wohl D. (2017). Bictegravir, emtricitabine, and tenofovir alafenamide versus dolutegravir, abacavir, and lamivudine for initial treatment of HIV-1 infection (GS-US-380-1489): A double-blind, multicentre, phase 3, randomised controlled non-inferiority trial. Lancet.

[31] Sax P.E., Pozniak A., Montes M.L., Koenig E., DeJesus E., Stellbrink H.-J., Antinori A., Workowski K., Slim J., Reynes J. (2017). Coformulated bictegravir, emtricitabine, and tenofovir alafenamide versus dolutegravir with emtricitabine and tenofovir alafenamide, for initial treatment of HIV-1 infection (GS-US-380-1490): A randomised, double-blind, multicentre, phase 3, non-inferiori. Lancet.

[32] Kityo C.M., Gupta S.K., Kumar P.N., Weinberg A.R., Gandhi-Patel B., Liu H., Hindman J.T., Rockstroh J.K. (2025). Efficacy and safety of B/F/TAF in treatment-naïve and virologically suppressed people with HIV ≥ 50 years of age: Integrated analysis from six phase 3 clinical trials. BMC Infect. Dis..

[33] Trizzino M., Pipitò L., Di Figlia F., Bonura S., Zimmerhofer F., Cicero A., Gioè C., Cascio A. (2025). Effectiveness and Safety of Bictegravir/Emtricitabine/Tenofovir Alafenamide in People Living with HIV Aged 50 Years and Older: A Retrospective Analysis of Naïve and Treatment-Experienced Individuals. Viruses.

[34] Troya J., Pousada G., Micán R., Galera C., Sanz J., de Los Santos I., Dueñas C., Cabello N., Martín C., Galindo M.J. (2024). Real-life data of immune recovery using bictegravir/emtricitabine/tenofovir alafenamide in virologically suppressed people living with HIV. Results at 48–96 weeks of RETROBIC Study. J. Antimicrob. Chemother..

[35] Maggiolo F., Rizzardini G., Molina J., Pulido F., De Wit S., Vandekerckhove L., Berenguer J., D’Antoni M.L., Blair C., Chuck S.K. (2023). Bictegravir/emtricitabine/tenofovir alafenamide in older individuals with HIV: Results of a 96-week, phase 3b, open-label, switch trial in virologically suppressed people ≥65 years of age. HIV Med..

[36] Serrano-Villar S., Deeks S.G. (2015). CD4/CD8 ratio: An emerging biomarker for HIV. Lancet HIV.

[37] Platt L., French C.E., McGowan C.R., Sabin K., Gower E., Trickey A., McDonald B., Ong J., Stone J., Easterbrook P. (2020). Prevalence and burden of HBV co-infection among people living with HIV: A global systematic review and meta-analysis. J. Viral Hepat..

[38] Inciarte A., Miralles C., Silva-Klug A., Revollo B., García Deltoro M., Portilla J., Antela A., Castaño Carracedo M.Á., De Álvaro C., Ramroth J. (2025). Effectiveness and Safety of Bictegravir/Emtricitabine/Tenofovir Alafenamide in People with HIV: A Real-World Data from the Spanish BICSTaR Cohort. HIV/AIDS-Res. Palliat. Care.

[39] Antinori A., Yokomaku Y., Elinav H., Pullukçu H., de Wet J., Antela A., Lu P.-L., Sabranski M., Kim Y.-S., Bonnet F. (2026). Quality of Life and Treatment Satisfaction in People with HIV Switching to Bictegravir/Emtricitabine/Tenofovir Alafenamide: Pooled Analysis from Observational Cohort Studies. Infect. Dis. Ther..

[40] Orkin C., Ajana F., Kityo C., Koenig E., Natukunda E., Gandhi-Patel B., Wang H., Liu Y., Wei X., White K. (2021). Brief Report: Efficacy and Safety of Bictegravir/Emtricitabine/Tenofovir Alafenamide in Females Living With HIV: An Integrated Analysis of 5 Trials. JAIDS J. Acquir. Immune Defic. Syndr..

[41] Kityo C., Hagins D., Koenig E., Avihingsanon A., Chetchotisakd P., Supparatpinyo K., Gankina N., Pokrovsky V., Voronin E., Stephens J.L. (2019). Switching to Fixed-Dose Bictegravir, Emtricitabine, and Tenofovir Alafenamide (B/F/TAF) in Virologically Suppressed HIV-1 Infected Women: A Randomized, Open-Label, Multicenter, Active-Controlled, Phase 3, Noninferiority Trial. JAIDS J. Acquir. Immune Defic. Syndr..

[42] Zhang H., Hindman J.T., Lin L., Davis M., Shang J., Xiao D., Avihingsanon A., Arora P., Palaparthy R., Girish S. (2024). A study of the pharmacokinetics, safety, and efficacy of bictegravir/emtricitabine/tenofovir alafenamide in virologically suppressed pregnant women with HIV. AIDS.

[43] Ambrosioni J., Levi L.I., Alagaratnam J., Sempere A., Mastrangelo A., Paioni P., Mussini C., Marzolini C., Nielsen S.D., Béguelin C. (2026). Major revision version 13.0 of the European AIDS Clinical Society guidelines 2025. HIV Med..

[44] Gazzola L., Tagliaferri G., De Bona A., Mondatore D., Borsino C., Bini T., Marchetti G., D’Arminio Monforte A. (2021). Dyslipidaemia after switch to tenofovir alafenamide (TAF)-based cART regimens in a cohort of HIV-positive patients: What clinical relevance?. HIV Med..

[45] Lacey A., Savinelli S., Barco E.A., Macken A., Cotter A.G., Sheehan G., Lambert J.S., Muldoon E., Feeney E., Mallon P.W. (2020). Investigating the effect of antiretroviral switch to tenofovir alafenamide on lipid profiles in people living with HIV. AIDS.

[46] Mallon P.W.G., Brunet L., Fusco J.S., Prajapati G., Beyer A., Fusco G.P., Wohlfeiler M.B. (2022). Lipid Changes after Switch from TDF to TAF in the OPERA Cohort: LDL Cholesterol and Triglycerides. Open Forum Infect. Dis..

[47] Martini S., Maggi P., Gervasoni C., Onorato L., Ferrara S., Alessio L., Bellacosa C., Esposito V., Di Filippo G., Masiello A. (2022). Dynamics of Lipid Profile in Antiretroviral-Naïve HIV-Infected Patients, Treated with TAF-Based Regimens: A Multicenter Observational Study. Biomedicines.

[48] Heseltine T., Hughes E., Mathew J., Murray S., Khoo S. (2022). The effect of changing to Bictegravir on lipids using real world data: A brief report. J. Clin. Pharm. Ther..

[49] Lim S.Y., Chin B., Kim M.-K., Kim G., Kim Y. (2025). Comparative analysis of lipid profile changes in treatment-naïve people living with HIV on INSTI-based single-tablet regimens, BIC/FTC/TAF and DTG/3TC: Real-world evidence from South Korea. J. Antimicrob. Chemother..

[50] Ciccullo A., Iannone V., Giacomelli A., Baldin G., Lagi F., Sambo M., Oreni L., Fabbiani M., Borghetti A., Papalini C. (2026). Changes in cardiovascular and metabolic risk scores after switching to DOR/3TC/TDF, DTG/3TC or BIC/FTC/TAF: Results from a multicenter Italian cohort. AIDS.

[51] García-Ruiz de Morales A.G., Suárez Robles M., Pérez-Elías M.J., Negredo E., Alcamí J., Gómez Rodríguez C.E., González-Ruano P., de Zárraga Fernández M.A., Dueñas Gutiérrez C., Moreno Guillén S. (2025). Metabolic Complications After Initiating BIC/FTC/TAF Versus DTG + 3TC in Antiretroviral-therapy-naive Adults With HIV: A Multicenter Prospective Cohort Study. Clin. Infect. Dis..

[52] Serrano-Villar S., Martín-Pedraza L., Tiraboschi J., Novella M., Cabello-Úbeda A., López-Cortés L., Busca C., Torralba M., Crusells M.J., Hidalgo-Tenorio C. (2026). Comparable Inflammatory and Metabolic Outcomes After Switching to Bictegravir/Emtricitabine/Tenofovir Alafenamide Versus Continuing Dolutegravir/Lamivudine in Virologically Suppressed Adults With HIV (INSTINCT/GESIDA10918 Study). Clin. Infect. Dis..

[53] Department of Health and Human Services Panel on Antiretroviral Guidelines for Adults and Adolescents. Guidelines for the Use of Antiretroviral Agents in Adults and Adolescents With HIV. https://www.ncbi.nlm.nih.gov/books/NBK586306/.

